# Enhanced U-Net with Multi-Module Integration for High-Exposure-Difference Image Restoration

**DOI:** 10.3390/s25041105

**Published:** 2025-02-12

**Authors:** Bo-Lin Jian, Hong-Li Chang, Chieh-Li Chen

**Affiliations:** 1 Department of Electrical Engineering, Chin-Yi University of Technology, Taichung 411030, Taiwan; bolin@ncut.edu.tw; 2 Department of Aeronautics and Astronautics, National Cheng Kung University, Tainan 701401, Taiwan; honglicool@gmail.com

**Keywords:** U-Net, high exposure difference, image restoration, dual attention unit, lightweight

## Abstract

Machine vision systems have become key unmanned vehicle (UAV) sensing systems. However, under different weather conditions, the lighting direction and the selection of exposure parameters often lead to insufficient or missing object features in images, which could fail to perform various tasks. As a result, images need to be restored to secure information that is accessible when facing a light exposure difference environment. Many applications require real-time and high-quality images; therefore, efficiently restoring images is also important for subsequent tasks. This study adopts supervised learning to solve the problem of images under lighting discrepancies using a U-Net as our main architecture of the network and adding suitable modules to its encoder and decoder, such as inception-like blocks, dual attention units, selective kernel feature fusion, and denoising blocks. In addition to the ablation study, we also compared the quality of image light restoration with other network models using BAID and considered the overall trainable parameters of the model to construct a lightweight, high-exposure-difference image restoration model. The performance of the proposed network was demonstrated by enhancing image detection and recognition.

## 1. Introduction

In recent years, drones have been widely used in various fields, such as the transportation of emergency medical needs or disaster relief in remote villages [1], air surveillance or geo-environmental monitoring [2], and construction and worker safety [3]. Especially in military applications, unmanned aerial vehicles can not only improve aerial combat capability for detecting and monitoring the enemy [4], but also provide instant and accurate information for timely fire support and the early planning of strikes and combat missions. However, visual information must be provided with quality under different environmental conditions so that subsequent image processing can be performed. For example, in hazy or high-exposure-difference (HED) environments, an improper choice of exposure parameters will affect the extraction of object features, impacting on the performance of target detection and autonomous driving systems, and even leading to a failure to perform specified tasks. Therefore, preprocessing the image data is crucial to the visual system.

Image restoration is receiving increasing attention in the field of computer vision. Regarding image exposure correction, most studies have focused on restoring low-light images to normal-light images. Wei et al. [5] proposed RetinexNet based on the Retinex theory, which considers an image as the product of the illumination and reflection components. RetinexNet enhances the details and clarity of the reflectance component in low-light images through denoising and improves the overall lighting effect by adjusting the brightness of the illumination component. Finally, the reflectance and illumination components are multiplied to obtain the restored image. Zamir et al. [6] introduced the MIRNet network, which utilizes a multi-scale feature map fusion method. It uses a dual-channel attention mechanism to select the feature information in the image and applies a selective kernel fusion mechanism to share information between different scales of feature maps and extract important features. These methods mainly focus on improving underexposed or overexposed images. Restoration methods for images with changes in light intensity can be categorized into three types: segmentation-based, fusion-based, and learning-based methods.

The segmentation-based method primarily splits lighting discrepancy images into backlight and frontlight regions and processes them separately. Zhenhao and Xiaolin [7] proposed an adaptive technique that handles backlight and frontlight regions by selecting their optimal tone mapping. Finally, the output images from both regions are fused to restore the lost details caused by the significant light differences. However, this method is time-consuming and requires more complex scenes, and the restoration effect may not be satisfactory because of the incomplete segmentation of the backlight and frontlight regions.

Wang et al. [8] proposed a multi-scale fusion method based on the fusion method to recover the HED images. This can improve the brightness and contrast of images. This method handles the dark and light regions of an image separately. It introduces a weight map to extract and estimate the details in the image and multiplies each image scale with the weight map to obtain the recovered image. However, the fusion method can produce color distortion and unnatural illumination in darker areas of the image. Buades et al. [9] used gamma and logarithmic tone mapping functions to improve the contrast of the backlit image for each color channel and used a modified Mertens’ algorithm [10] to fuse the images and further optimize the quality of the fused image by sharpening the details and correcting the chromaticity.

Li et al. [11] introduced an event-based high-dynamic-range imaging (EHDRI) framework that adaptively fuses single-exposure standard-dynamic-range (SDR) images with concurrent events for HDR reconstruction. The framework incorporates an Exposure Attention Fusion (EAF) module for adaptive feature fusion under various exposure conditions. It implements the enhance details and decrease noise self-supervised loss for simultaneous detail enhancement and noise reduction. For overexposed regions, the system primarily relies on the high-dynamic-range information captured by event cameras, with the EAF module adaptively controlling the fusion ratio between SDR image and event features.

Jia et al. [12] presented an ultra-high-definition multi-exposure image fusion method that leverages a U-shaped network structure for multi-scale feature extraction. The framework incorporates a hybrid stacking paradigm combining CNN modules with Transformer modules to capture local texture features and global color features simultaneously. The system employs a self-attention convolution module for handling overexposed regions to achieve adequate local representation learning while maintaining long-range dependencies. The method introduces a cross-layer feature fusion module that adaptively fuses layered features through learnable correlations between different layers.

Lin et al. [13] introduced a dual-domain feature learning model for nighttime semantic segmentation, incorporating three key components: exposure attention correction module, frequency domain transformation module, and dual-domain cross-fusion module. U-Net is utilized in the decoding block to generate segmentation maps and fuse low- and high-level features, preserving high-resolution details. The architecture effectively combines traditional spatial domain analysis with frequency domain processing to capture global contextual information and detailed local features. Through extensive experiments across various network backbones and datasets, the research validates that their dual-domain feature learning framework significantly improves nighttime semantic segmentation performance while maintaining computational efficiency. The methodology explicitly addresses exposure-related challenges in segmentation tasks, where traditional approaches often struggle with varying lighting conditions.

Yaqoob et al. [14] proposed a design explicitly for deep learning-based fracture segmentation in geological outcrops. U-Net is a key validation architecture achieving an IoU score of 85% and a pixel accuracy of 92%. The segmentation methodology incorporates spatial and frequency domain features through a dual-domain feature learning model, effectively addressing challenges in nighttime image processing and complex exposure patterns. U-Net is implemented in the decoding block of their architecture to generate segmentation maps and fuse low- and high-level features while maintaining high-resolution details, demonstrating its efficacy in fracture edge detection tasks. The dataset addresses real-world challenges in fracture edge segmentation, including contrast variations, exposure issues, and physical occlusions, providing a comprehensive benchmark for developing and evaluating segmentation algorithms. The study’s success in achieving high accuracy scores demonstrates the potential of deep learning approaches in automated geological feature segmentation, significantly when leveraging carefully curated datasets and appropriate neural network architectures.

With the rapid development of computer technology, the performance of Graphics Processing Units (GPUs) dedicated to handling graphics computations has significantly increased. In recent years, deep learning methods have greatly benefited from the parallel processing capabilities of GPUs. However, owing to the lack of large-scale datasets for HEDs, there are few learning-based HED restoration methods. Some of these methods include Zhang et al. [15], who used zero-shot learning to restore the lost details caused by HEDs. The study [15] also used a small specific dataset to train a neural-like network called the Exposure Correction Network (ExCNet) to estimate the S-curve suitable for backlit test images. Once the S-curve is estimated, it can be directly applied to restore test images. In addition, Lv et al. [16] proposed the BAcklit Image Dataset (BAID) because there is no large-scale HED dataset at that stage, and the lack of real images in the existing datasets hinders the application of end-to-end learning on the network.

In recent years, owing to the excellent performance of U-Net [17] in the field of image segmentation, many scholars have applied U-Net or modified versions of it for various image recovery tasks. Furthermore, considering the goal of achieving model light weighting, Tsai and Chen [18] used inception-like blocks to significantly reduce the overall training parameters in the image dehazing. Their study aimed to provide a lightweight neural network architecture capable of restoring images with HEDs. The proposed network was designed to meet the efficiency requirements of real-time applications while emphasizing the model’s effectiveness.

As highlighted in the literature review above, existing approaches to high-exposure-difference image restoration can be categorized into segmentation-based, fusion-based, and learning-based methods. While segmentation-based methods like adaptive tone mapping show promise, they often struggle with complex scenes and incomplete region segmentation. Fusion-based approaches have demonstrated improvements in brightness and contrast but frequently suffer from color distortion in darker regions. Recent learning-based methods, particularly those leveraging deep neural networks, have shown encouraging results but are often constrained by computational complexity or the lack of large-scale training datasets. To address these limitations, the main contribution of this work is the development of a lightweight neural network architecture for high-exposure-difference image restoration (HEDIR) based on an enhanced U-Net framework. Our proposed method incorporates several key innovations: (1) the integration of inception-like blocks that efficiently extract multi-scale features while significantly reducing computational complexity; (2) the implementation of dual attention units (DAUs) and selective kernel feature fusion (SKFF) mechanisms that adaptively focus on relevant features in both spatial and channel dimensions; (3) the addition of denoising blocks to enhance image clarity while preserving structural details; and (4) the design of a combined loss function utilizing both structural similarity (SSIM) and mean absolute error (MAE) metrics to optimize restoration quality. The remainder of this paper is organized as follows: Section 2 provides the theoretical background of high-exposure-difference image restoration and neural networks. Section 3 presents our proposed method, including the network architecture and its key components. Section 4 demonstrates the experimental results, including comprehensive ablation studies, performance comparisons with state-of-the-art methods, and practical applications in object detection tasks. Finally, Section 5 concludes the paper with a summary of our findings and potential future directions.

## 2. High-Exposure-Difference Image Restoration Neural Networks

Deep learning methods can utilize the correspondence between the original image and the target image to construct a network model and restore the lost details owing to the high exposure difference in the image. However, using a deep learning method does not simply mean increasing the network size or number of layers to achieve better restoration results. Simply deepening the deep learning model will lead to longer training and execution time, which may not meet the requirement of quickly restoring the HED in the images. Therefore, when using deep learning methods, it is essential to explore the overall convolutional neural network (CNN) architecture and appropriate network modules to effectively recover HED images.

### 2.1. Downsampling and Upsampling

Downsampling and upsampling are used to reduce and increase the resolution of the original image by a specified factor, respectively. In general, downsampling involves max-pooling to retain the values of the most prominent feature maps. The degree of downsampling was adjusted by setting the pooling kernel size and stride. Max-pooling is a predefined operation that improves the receptive field without increasing computational complexity. Upsampling typically uses predefined interpolation methods such as nearest-neighbor interpolation and bilinear interpolation. Therefore, using convolution and transposed convolution allows the CNN to know the appropriate downsampling and upsampling methods during training. For example, to reduce the image resolution to half that of the original image, convolution with a stride of two can be used instead of max-pooling to avoid sacrificing the values of other feature maps. To increase the image resolution to twice that of the original image, transposed convolution with a stride of two can be used instead of the interpolation algorithm.

In addition, when using transposed convolution, because the kernel size is not divisible by the stride, uneven overlap may occur, leading to artifacts such as the checkerboard effect [19]. Therefore, the transposed convolutions used in this study had a stride of two and a kernel size of two to avoid creating similar checkerboard-like patterns in the output images.

### 2.2. U-Net-Based Image Convolutional Neural Network

The proposed CNN architecture for HED image restoration was based on U-Net [17] as a prototype. U-Net is composed of two main parts: an encoder and a decoder. The encoder comprises multiple convolution filters and max-pooling, which downsample the image and reduce the image size to extract features. The decoder comprises multiple convolutions and transposed convolutions to upsample an image. Before the decoder performs convolution in the same layer of the encoder and decoder, the feature maps of the same spatial size generated by the same layer of the encoder are concatenated with the feature maps of the previous layer by transposed convolution as the input of the convolution. Combining the upsampling and downsampling operations allows the model to effectively capture image details from different spatial dimensions, leading to more accurate image segmentation.

U-Net is primarily used for image segmentation tasks in a U-Net-based network architecture. Therefore, to perform other image processing, it is necessary to introduce appropriate network modules based on the characteristics of a specific task rather than relying solely on the scale and depth of the network architecture. For example, in an image-dehazing task, Tsai and Chen [18] proposed an image-dehazing CNN based on U-Net. In this modified U-Net, the authors incorporated an attention mechanism before performing convolutions in each corresponding layer of the encoder and decoder. This attention mechanism automatically focuses on useful features in an image and suppresses irrelevant features, resulting in clearer dehazed images. Additionally, they replaced the conventional convolutions in the encoder and decoder with inception-like blocks to improve computational efficiency during network training.

Because U-Net has demonstrated good performance in various image restoration tasks in recent years, including enhancing image quality, this study adopted U-Net as the prototype of the basic architecture in HEDIR. Various network modules, such as inception-like blocks, dual attention units, selective kernel feature fusion (SKFF), and denoising blocks, were incorporated into U-Net to develop an effective HEDIR model.

## 3. Proposed Method

**Figure 1:**
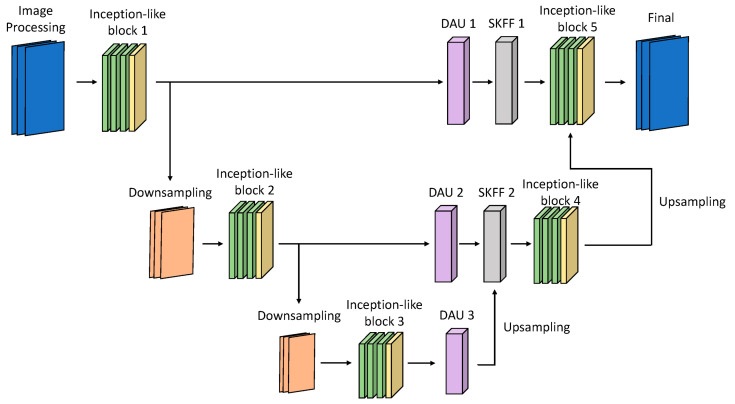
The proposed CNN architecture for image restoration with high exposure difference.

Figure 1 illustrates the proposed HEDIR-CNN based on U-Net architecture. Because the original U-Net architecture using max-pooling for downsampling results in the loss of feature map details, max-pooling is replaced with convolution layers with a stride of two. The input images must be normalized before processing using HEDIR-CNN. Normalization scales the pixel values of the images within a specific range, eliminating the issue of significant differences in the pixel values. This accelerates the gradient descent during model training and enhances the results after training. In this study, min-max normalization is employed, as shown in Equation (1), to linearly transform the pixel values of the images into the range [0, 1] before inputting them into HEDIR-CNN.

(1) $$L_{c}^{n}(x)=\frac{L_{c}(x)-\min(L_{c}(x))}{\max(L_{c}(x))-\min(L_{c}(x))}$$

where is x the pixel position in the image, $L_{c}(x)$ is one of the color spaces of the HED image L(x), $L_{c}^{n}(x)$ is the normalized image, and c∈{R,G,B} represents the red, green, and blue color channels; $\min(L_{c}(x))$ means that the minimum value is taken in the c-channel L(x), and, similarly, $\max(L_{c}(x))$ means that the maximum value is taken in the c-channel L(x).

In this study, HEDIR-CNN replaces the step of concatenating U-Net feature maps along the channel axis with dual attention mechanisms and SKFUs. These modifications adjust the gain of feature maps and combine an image-denoising block to enhance image quality. The following sections introduce the modules used in detail.

### 3.1. Inception-like Block

Convolution is used to extract image features and form feature maps. These feature maps represent high-dimension images. The feature maps obtained by the convolutional filter are usually convolved and then passed through non-linear operations, such as the Rectified Linear Unit (ReLU) or sigmoid function, to increase the non-linear features of the neural network.

**Figure 2:**
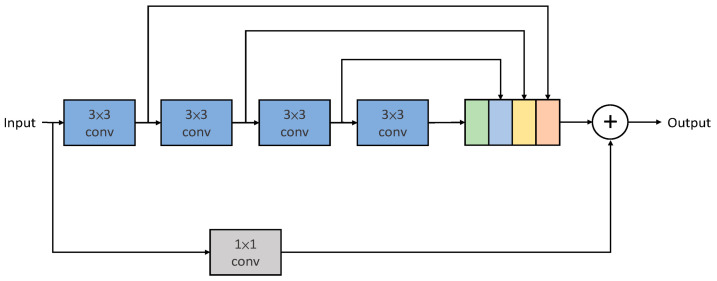
Inception-like block [14].

Szegedy et al. [20] proposed a novel network convolution structure and introduced the concept of decomposing convolution layers, in which a large convolution layer is decomposed into two or more smaller convolution layers. For example, a 5 × 5 convolution kernel is replaced with two 3 × 3 convolution kernels. In this case, the number of parameters of a 5 × 5 convolution is 2.78 (25/9) times that of one 3 × 3 convolution. Therefore, using two 3 × 3 convolutions to replace one 5 × 5 convolution can reduce the number of parameters by several and decrease the computational workload by approximately 28%. Similarly, a 7 × 7 convolution kernel has 5.44 (49/9) times the number of parameters as a convolution. Thus, using three 3 × 3 convolutions to replace one 7 × 7 convolution can reduce the number of parameters to 0.55 (27/49) times and decrease the computational workload by approximately 45%. By decomposing the convolution layers and using multiple smaller convolution layers to replace larger ones, we can effectively reduce the number of training parameters and improve the computational efficiency. Tsai and Chen [18] concatenated the outputs of the input image using convolutional kernels of varying sizes, including 3 × 3, 5 × 5, and 7 × 7. They also utilized a 1 × 1 convolutional kernel as a residual input to enhance feature extraction capabilities using diverse receptive fields while reducing the number of network training parameters, thus further improving training efficiency, as shown in Figure 2.

### 3.2. Dual Attention Unit

**Figure 3:**
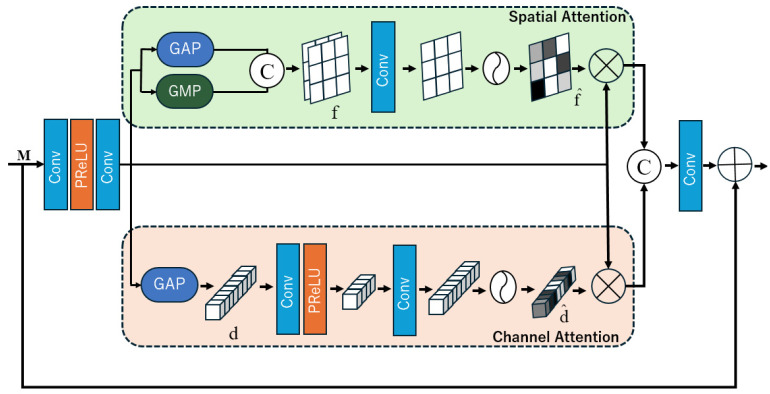
Dual attention unit [6]. C denotes the concatenation operation. d is the feature descriptor. $\hat{d}$ generates activations. M, and f is the feature map. $\hat{f}$ is the spatial attention map.

In the original U-Net [17], feature fusion involves directly concatenating the output feature maps of the encoder at the same layer with the output of the previous transposed convolution. In Attention U-Net [21], this concatenation is replaced by attention gates, in which the outputs from the same layer encoder and the output of the previous transposed convolution are fed into the attention gate. Feature maps of the same dimensions are multiplied by attention coefficients ranging between 0 and 1, effectively assigning pixel-specific weight values through attention coefficients to suppress irrelevant regions in the image. MIR-Net [6] introduced dual attention units (DAUs) to address the limitation of previous attention mechanisms, which considered only a single dimension for learning important features in an image. DAUs consist of two attention mechanisms, channel attention (CA) and spatial attention (SA), as shown in Figure 3. The feature maps from CA and SA are concatenated and preserved through residual connections with the input feature map M. By employing DAUs, the model can not only share information and compensate for the features overlooked by one dimension but also help suppress irrelevant features. This allows only the most informative features to be propagated to deeper network layers.

Spatial Attention: The spatial attention mechanism aims to extract spatial features from the convolutional feature map and generate a spatial attention map. Based on this attention map, the original input feature map M is re-adjusted to focus on the relevant spatial regions in the image, thereby enhancing the effectiveness of image training.

Channel Attention: The channel attention mechanism aims to extract the features from different channels of the convolutional feature map to generate a channel attention map. Based on this attention map, the original input feature map M is adjusted by focusing on the important features presented in each channel, such that the negative impact due to irrelevant feature channels is reduced.

### 3.3. Selective Kernel Feature Fusion

**Figure 4:**
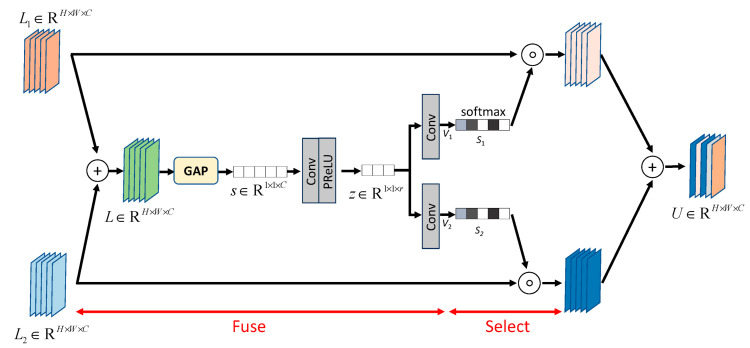
Selective kernel feature fusion [6].

In typical convolutional neural networks, the receptive field size for each neuron is fixed, which may limit the amount of information that can be extracted. When the receptive field size is larger than the region features to be extracted, it may treat parts of the image as the background and fail to capture its features. However, if the receptive field size is smaller than the region of features to be extracted, it may introduce excessive local information and weaken the essential information. Jian et al. [22] proposed the selective kernel unit (SKU) that allows the network to change the receptive field size during feature extraction to capture more comprehensive feature information. Zamir et al. [6] introduced the selective kernel fusion (SKF) method inspired by [22]. Traditional feature extraction methods, such as simply using a fixed-size convolutional layer, can provide only limited feature information. However, SKFU considers different scales of feature maps and uses them as the inputs. It employs multi-scale feature generation within the same layer and automatically adapts the optimal receptive field size through feature fusion and selection, as illustrated in Figure 4. In the figure, + represents element-wise summation, ○ denotes element-wise multiplication, and GAP represents global average pooling. Feature fusion and selection processes are described as follows:

1. As illustrated in Figure 4, SKFU uses the feature maps obtained from the DAUs in the same layer and the output from the previous transposed convolutional layer as inputs. It performs an element-wise addition between these two feature maps to obtain the fused feature map L.
2. The feature map L undergoes global average pooling along the spatial dimension to obtain channel-wise statistics, denoted as s. Then, s is used as the input for a channel-wise downsampling convolutional layer to obtain the compact feature representation z. This process reduces the original feature channels’ dimension and generates a compact feature representation, improving computational efficiency.
3. Finally, the compact feature representation passes through two parallel channel-wise upsampling convolutional layers, resulting in two feature descriptors $v_{1},\;v_{2}\in R^{1\times 1\times C}$.

Feature Fusion:

1. As illustrated in Figure 4, the fusion operation learns different feature descriptors $v_{1},v_{2}$ for each channel through a learning process. The softmax function is then used to scale the weights of each channel between 0 and 1, obtaining attention activation $s_{1},s_{2}\in R^{1\times 1\times C}$.
2. The attention activation is multiplied element-wise with the original input feature map $L_{1}$ and $L_{2}$. After re-calibration and aggregation, the resulting representation of the output feature maps can be expressed as Equation (2):
  (2) $$U=L_{1}\cdot s_{1}+L_{2}\cdot s_{2}$$

### 3.4. Denoising Block

Image denoising is a very important field in image processing, which aims to remove noise from images and obtain clearer visual results. Gurrola-Ramos et al. [23] proposed a Residual Dense U-net (RDUNet) for image denoising. RDUNet also uses U-net [17] as its main network architecture, by adding denoising blocks at the end of the encoder and decoder. Denoising blocks are composed of a series of densely connected convolutional layers, considering all the feature maps outputted by the layers within the block. Using residual connections accelerates learning and helps avoid the vanishing gradient problem. In this paper, the proposed HEDIR-CNN references the denoising block from RDUNet, which can be added to each layer of the encoder and decoder to enhance image quality further.

**Figure 5:**
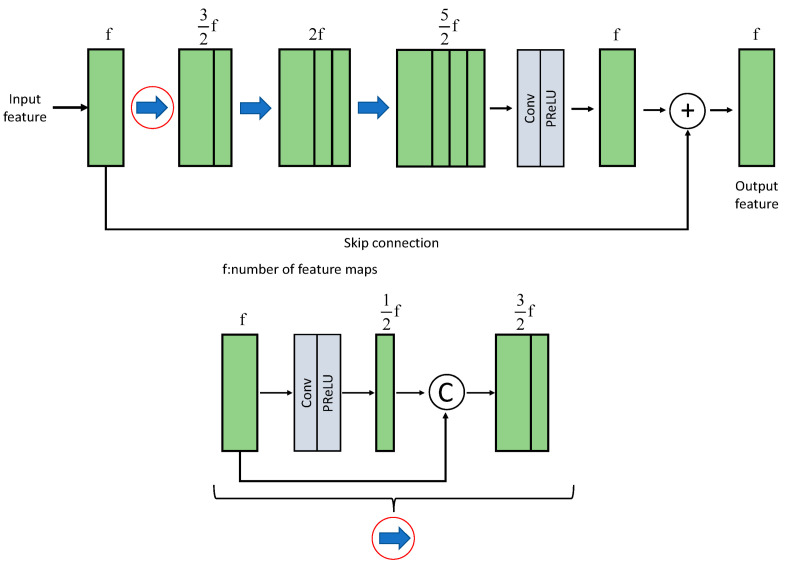
Denoising block.

The image denoising blocks utilize dense blocks and residual connections. The dense block comprises multiple 3 × 3 convolutional layers, where each layer’s output is concatenated with the previous layers, enabling the repeated consideration of previous feature maps. The input feature map first passes through a 3 × 3 convolutional layer to reduce its channel dimensions. It is then combined with the original input feature map and fed into the next convolutional layer. This process is repeated iteratively. Finally, a 3 × 3 convolutional layer integrates all previous feature maps along with the input to the denoising block. The output feature map from this convolutional layer has the same number of channels as the input to the denoising block, thereby conducting residual connections. The residual connections allow the network to use previous features during forward propagation and avoid gradient vanishing problems that may arise from deepening the network architecture. Figure 5 illustrates the process, where the blue arrows represent the concatenation of the input and output feature maps after the convolution.

### 3.5. Loss Function

The loss function used in this study is the structural similarity index measure (SSIM) [24,25] represented by Equation (3):

(3) $$SSIM(P)={\left[l(x,y)\right]}^{\alpha }\cdot {\left[c(x,y)\right]}^{\beta }\cdot {\left[s(x,y)\right]}^{\gamma }$$

where *P* is the patch, and l(x,y), c(x,y), and s(x,y) are the luminance similarity index, contrast similarity index, and structure similarity index, respectively. *x* in (x,y) represents the output of the CNN for a restored image with a HED, and y represents a clear image. In this formula, α, β, γ are typically set to 1. The quality of image restoration can be improved by learning the similarity between the restored image and the ground-truth image in terms of luminance, contrast, and structural information. A higher SSIM value closer to 1 indicates a greater similarity between the two images.

The formulas for the luminance similarity index, contrast similarity index, and structure similarity index are given in Equation (4).

(4) $$\left\{\begin{array}{l} l(x,y)=\frac{2\mu_{x}\mu_{y}+C_{1}}{\mu_{x}^{2}+\mu_{y}^{2}+C_{1}}\\ c(x,y)=\frac{2\sigma_{x}\sigma_{y}+C_{2}}{\sigma_{x}^{2}+\sigma_{y}^{2}+C_{2}}\\ s(x,y)=\frac{\sigma_{xy}+C_{3}}{\sigma_{x}\sigma_{y}+C_{3}} \end{array}\right.$$

where $\mu_{x}$ and $\mu_{y}$ in l(x,y) denote the mean values of x and y, respectively. $\sigma_{x}$ and $\sigma_{y}$ in c(x,y) denote the standard deviations of x and y, respectively. $\sigma_{xy}$ in $s\left(x,y\right)$ denotes the covariance of x and y. $C_{1}$, $C_{2}$, and $C_{3}$ are constants.

In addition to using SSIM as the loss function, this study also utilized the mean absolute error (MAE) as another loss function, represented by Equation (5). MAE is used as the lost function to enable the network to focus mainly on the pixel-wise differences between the restored image and the clear image during the learning process.

(5) $$L_{MAE}(P)=\frac{1}{N}\sum_{p\in P}\left|x(p)-y(p)\right|$$

where N is the total number of pixels in the patch.

Compared to the mean squared error (MSE), which is also known as $L_{2}$ loss function and is commonly used as a loss function, Zhao et al. [24] pointed out that using MSE can excessively magnify the impact of large errors and affect convergence during model training. Additionally, artifacts are more likely to be generated after restoration in areas of the image where there are no objects or other structures. Considering the presence of similar regions in the HED images, MAE was adopted as the loss function.

In this study, the loss function was a combination of SSIM and MAE, as shown in Equation (6). This combined loss function considers the pixel-wise differences between the output of HEDIR-CNN and the clear image and the relational differences between pixels.

(6) $$Loss=(1-\alpha )\cdot L_{SSIM}+\alpha \cdot L_{MAE}$$

where α = 0.2 is referred to the experiments of Liu and Zhao [26] and Zhao et al. [24], while both *L_SSIM_* and *L_MAE_* are normalized owing to their own definition and the normalized pixel value, respectively.

## 4. Experiments and Analysis

In this section, the proposed HPDIR-CNN is applied to conduct image-restoration experiments and demonstrate the effectiveness of the designed CNN model. To meet the requirement of a lightweight model, we first confirmed that the various parameters in the HEDIR-CNN architecture, such as different sizes of convolutional kernels, were employed in various network modules to obtain the training parameters within the model. Next, we introduced the high-exposure-difference dataset used for training the HEDIR-CNN model, and then input the test dataset to the trained HEDIR-CNN to evaluate the image restoration performance and compare it with other model methods. The restored images were further used for subsequent object recognition tasks to validate the performance of the designed HEDIR-CNN. Additionally, the performance of the proposed CNN was investigated for images under different weather conditions. The results of HEDIR-CNN are presented below.

### 4.1. Parameters of Neural Network

Considering the overall number of trainable parameters and the image restoration time, it is essential to strike a balance between increasing the trainable parameters to improve image restoration quality and maintain a lightweight network and fast restoration speed. To achieve this, the image input to HEDIR-CNN was standardized with dimensions of 512, and according to Equation (1), the RGB image was formed with a channel size of 3. Thus, the input image dimensions were 3 × 512 × 512. The HEDIR-CNN architecture designed in this study includes five inception-like blocks, three DAUs, two SKFUs, five denoising blocks, two downsampling, and two upsampling layers. The total number of trainable parameters is approximately 1.735 million. Table 1 summarizes the internal neural network structure of HEDIR-CNN for image restoration.

**Table 1:** Internal neural network structure of the proposed network.

Blocks in Figure 1	Layers	Num	Blocks in Figure 1	Layers	Num
Inception-like Blocks 1 and 5	Conv2D(3,3) Conv2D(3,3) Conv2D(3,3) Conv2D(3,3) Conv2D(1,1)	8 8 8 8 32	Selective Kernel Feature Fusion 1	Conv2D(1,1) Conv2D(1,1) Conv2D(1,1)	4 32 32
Inception-like Blocks 2 and 4	Conv2D(3,3) Conv2D(3,3) Conv2D(3,3) Conv2D(3,3) Conv2D(1,1)	16 16 16 16 64	Selective Kernel Feature Fusion 2	Conv2D(1,1) Conv2D(1,1) Conv2D(1,1)	8 64 64
Inception-like Block 3	Conv2D(3,3) Conv2D(3,3) Conv2D(3,3) Conv2D(3,3) Conv2D(1,1)	32 32 32 32 128	Denoising Blocks 1 and 5	Conv2D(3,3) Conv2D(3,3) Conv2D(3,3) Conv2D(3,3)	16 16 16 32
Dual Attention Unit 1	Conv2D(5,5) Conv2D(3,3) Conv2D(3,3) Conv2D(1,1) Conv2D(1,1) Conv2D(1,1)	1 32 32 4 32 32	Denoising Blocks 2 and 4	Conv2D(3,3) Conv2D(3,3) Conv2D(3,3) Conv2D(3,3)	32 32 32 64
Dual Attention Unit 2	Conv2D(5,5) Conv2D(3,3) Conv2D(3,3) Conv2D(1,1) Conv2D(1,1) Conv2D(1,1)	1 64 64 8 64 64	Denoising Block 3	Conv2D(3,3) Conv2D(3,3) Conv2D(3,3) Conv2D(3,3)	64 64 64 128
Dual Attention Unit 3	Conv2D(5,5)	1	Downsampling 1	Conv2D(2,2)	32
	Conv2D(3,3)	128	Downsampling 2	Conv2D(2,2)	64
	Conv2D(3,3)	128	Upsampling 1	ConvT2D(2,2)	32
	Conv2D(1,1)	16	Upsampling 2	ConvT2D(2,2)	64
	Conv2D(1,1)	128	Final convolution	Conv2D(1,1)	3
	Conv2D(1,1)	128			

### 4.2. High-Exposure-Difference Dataset

Training neural networks requires a large and high-quality dataset to help them extract important features from images during training. However, constructing a large-scale training dataset is challenging because obtaining both HED images and ground-truth images in the same scene is difficult. Moreover, to ensure the robustness of the network, the dataset must encompass various scenes. Therefore, considering these factors, this study adopted BAID built by the authors of [16] as the training dataset for HEDIR-CNN. BAID was developed to address the current lack of large-scale high-exposure-difference datasets and the absence of real-world images in existing datasets, which limit their applicability for end-to-end learning in network training. Their approach involved the use of different camera models to capture HED images. These captured images were then handed over to five photography experts, who used image editing software to enhance them. Finally, 20 volunteers were invited to rate the five edited versions of each image, and the highest-rated version was chosen as the ground-truth image.

**Figure 6:**
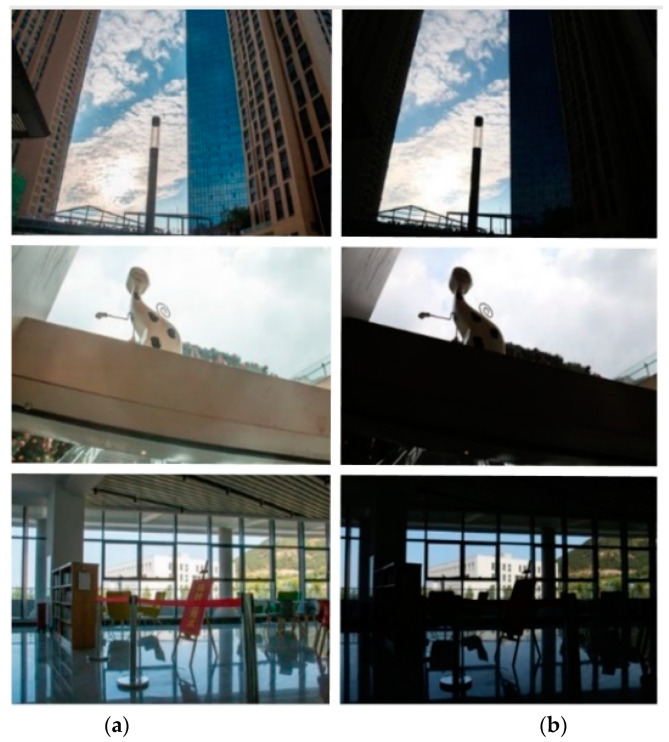
Randomly selected images of HED images and corresponding clear images from BAID. (**a**). Clear images; (**b**) HED images.

BAID consisted of a total of 3000 HED images along with their corresponding ground-truth images. The dataset included a wide range of scenes, themes, weather conditions, and diverse lighting conditions, covering both indoor and outdoor environments, as illustrated in Figure 6 and summarized in Table 2. The dataset was then randomly divided into a training set of 2600 images and a test set of 400 images. The image sizes were 5472 × 3648 and 3648 × 5472.

This study used 90% of the BAID training dataset to train the HEDIR-CNN model, whereas the remaining 10% was utilized for model testing. The quality metrics for the restored images are calculated using the BAID test dataset.

### 4.3. Ablation Study

This study used PyTorch 1.12.1 to construct a deep learning network for HEDIR-CNN. The processor used is Intel Core i7-12700 CPU 2.1 GHz, and the training was performed on the image processor GeForce RTX 3090.

In this section, we explore the importance of each network module in the proposed HEDIR-CNN. To better understand the design and performance of the model, we modified the model to test the impact of each network module. The purpose of the ablation experiments was to verify the effectiveness of each added network module in terms of the quality of recovery. In this study, six different models were trained to investigate the performance of these modules; the six models are summarized in Table 3. In the table, “-” represents the absence of the corresponding network module, “○” indicates the usage of the network module, and Model 6 represents the proposed HEDIR-CNN designed in this study.

During testing, the BAID test dataset was used to calculate the Peak Signal-to-Noise Ratio (PSNR) and SSIM metrics. PSNR is a measure of image quality, given by Equation (7). It evaluates the difference in pixel values between the degraded image and the reference clear image, comparing the two quantitatively. Higher PSNR values indicate better image quality [27].

(7) $${\mathrm{PSNR}=20\log}_{10}(\frac{\mathrm{Max}}{\sqrt{\mathrm{MSE}}})$$

In this equation, Max represents the maximum pixel value in the image. For an 8-bit image, Max = 255. Table 4 provides the six models’ HEDIR metrics and their corresponding trainable parameter counts.

The ablation experiment results demonstrate that the employed inception-like blocks, DAUs, SKFUs, and denoising blocks are all essential network components, significantly improving both PSNR and SSIM. From Table 4, we can observe that Models 1 and 2, with the addition of inception-like blocks, exhibit enhanced HEDIR quality while reducing overall trainable parameters. Model 4, incorporating DAUs, further improved the SSIM and PSNR compared to Model 3. Additionally, Model 5, which includes SKFUs, reduces trainable parameters and enhances the quality of the recovered images compared to Model 4. Finally, Model 6, the proposed HEDIR model, utilized all network modules and achieved the best recovery quality among all tested models.

### 4.4. Performance and Discussions

The BAID training dataset was trained for 400 epochs with a batch size of 6. The loss function used is shown in Equation (6). The input image size for HEDIR-CNN was 512 × 512. During training, 90% of the dataset was used for training, and the remaining 10% was used for validation. The entire network was optimized using the Adam optimizer [28], with a learning rate of 1 × 10^−4^.

After training the model, the results of the high-exposure-difference recovery image were analyzed and compared using two common image evaluation metrics: PSNR and SSIM. Table 5 shows a comparison between the proposed method and three other HEDIR recovery methods organized by [16]. The comparison involved applying different methods to the BAID test dataset for image restoration evaluation. From Table 5, we can see that the proposed CNN for HEDIR exhibits significant improvements in both image quality metrics.

Then, a further assessment was conducted to evaluate the average execution time required for the designed HEDIR CNN to process an image from input to restoration. In total, 400 HED images with a resolution of 512 × 512 were used to estimate the execution time required to recover an image by the proposed HEDIR-CNN. The results show that it only requires approximately 0.0816 s on average to restore one image, which is faster than the human reaction time of 0.1 s and reduces the time by about 20%.

**Figure 7:**
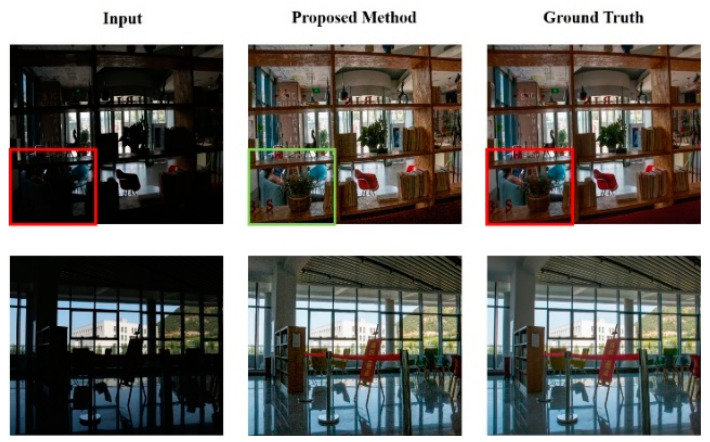
Before-and-after indoor image restoration in the BAID test dataset.

After discussing the results of the image metrics, Figure 7 illustrates a comparison between the images restored by the proposed CNN and their corresponding clear images from the BAID test dataset. This figure presents a comparative illustration of indoor HED images before and after restoration, showing that the restored images are almost indistinguishable from the clear images. Moreover, in areas where the original images are dark owing to significant light disparity, the objects become clearly identifiable after being restored by HEDIR-CNN. For example, in the first column of the figure, the person and the objects inside the box are discernible in the restored image.

**Figure 8:**
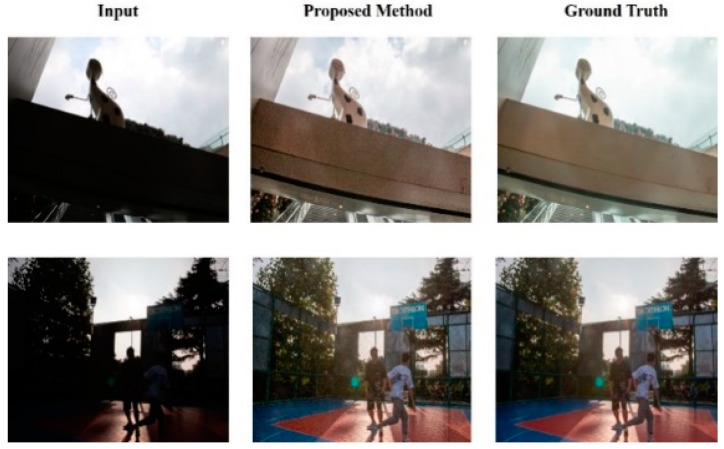
Before-and-after outdoor image restoration in the BAID test dataset.

Figure 8 compares the image before and after the recovery of light disparity in the outdoor area. When shooting outdoors, important details in images can be lost owing to excessive background lighting or the sun being positioned behind, causing people or objects to appear too dark. However, after restoration using the proposed method, the structures and details of the previously unidentifiable content in the image are significantly improved.

**Figure 9:**
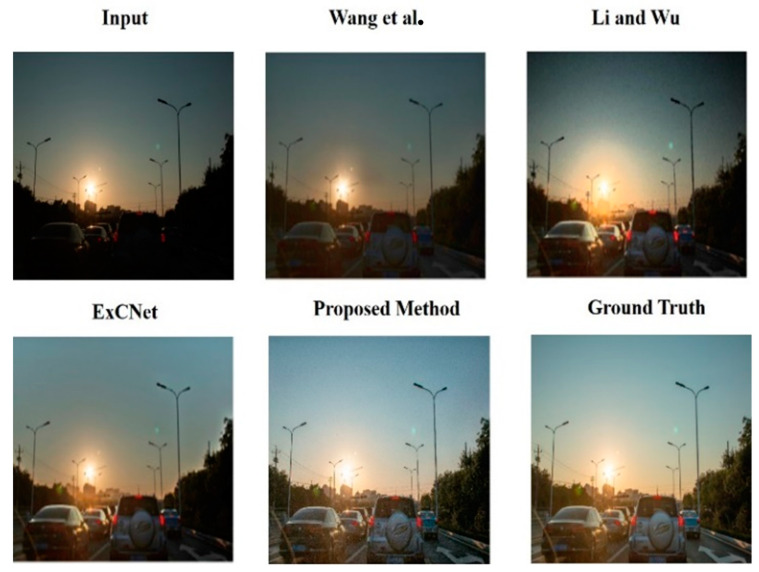
Comparison of restoration results [25].

Figure 9 shows the comparison results between those provided by [16] and those restored using the proposed image method. Wang, Fu, Zhang and Ding [8] only slightly enhanced the darkened areas caused by light disparity on the road surface. Zhenhao and Xiaolin [7] and Zhang, Zhang, Liu, Shen, Zhang and Zhao [15] improved the contours and structures of cars to some extent, but the overall brightness was unsatisfactory. In contrast, our proposed method effectively restores the image, clearly identifying the contours and structures of cars and road markings.

**Figure 10:**
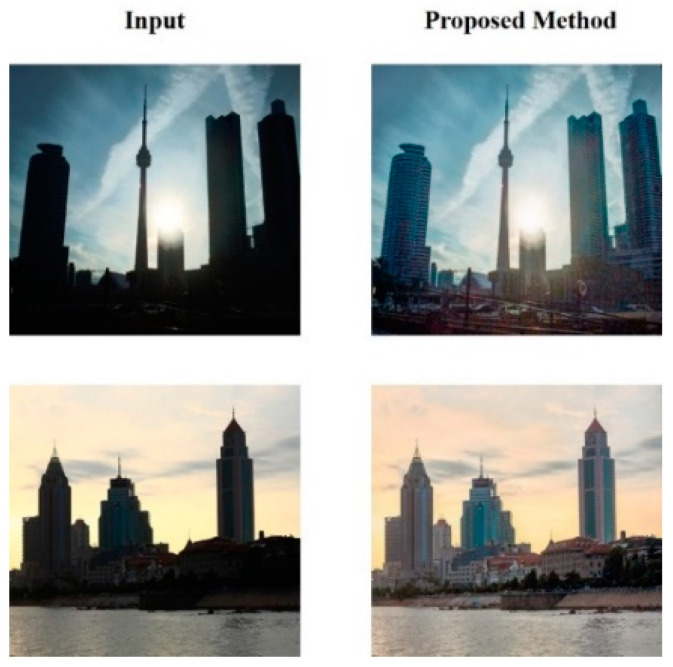
Before-and-after image restoration.

From Figure 7, Figure 8 and Figure 9, it is evident that the proposed HEDIR-CNN outperforms other light restoration methods on the BAID test dataset, achieving superior image restoration results. After achieving satisfactory restoration results on BAID, the designed network was expected to exhibit similarly good restoration capabilities in a wider range of scenarios. For this purpose, we used the HED dataset proposed by [7] for validation. Figure 10 illustrates the results after restoration using the proposed HEDIR-CNN, in which the detail loss caused by light disparity was recovered effectively. This further confirmed the good generalization capability of the proposed method.

### 4.5. Image Recognition

Image recognition often follows image restoration as a secondary task, where the restored image from the previous stage is provided for object recognition in this stage. After verifying the considerable restoration capability of the designed HEDIR-CNN in the previous sections, we further tested the performance of the designed neural network in the image recognition stage and observed an improvement in target recognition after image recovery. This section focuses on validating the performance of the designed HEDIR-CNN. The image recognition stage was primarily used to confirm the benefits of the previous stage. Therefore, we used a simple method for object detection. The restored images from the damaged inputs were provided for the subsequent object recognition task.

In this study, image recognition was performed using YOLOv4 (You Only Look Once) [29], a classic object detection method in the field of image recognition. YOLOv4 is a single-network design that can determine the type and position of target objects, making it an easily trainable and fast method that significantly improves recognition speed.

**Figure 11:**
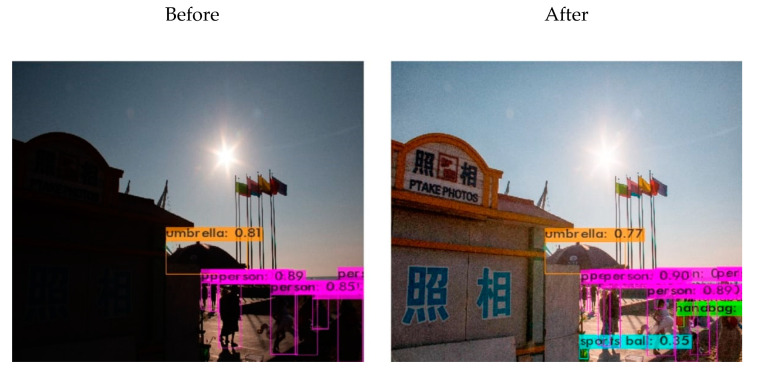
Object detection before and after restoration.

**Figure 12:**
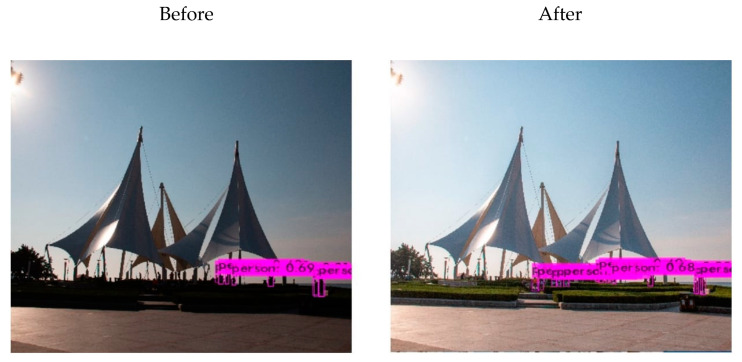
Additional object detection before and after restoration.

The results of human detection using YOLOv4 on the restored images obtained by the proposed method are presented in Figure 11, from which the number of persons detected was increased from 8 to 13. Similarly, Figure 12 illustrates an object detection comparison of another set of before-and-after image restoration, and more information is provided in Table 6. After object detection and recognition, the proposed restored image allows for the identification of more people and enables the recognition of additional objects, such as balls and bags. The experimental results indicate that the images recovered by the proposed network exhibit improved performance in object recognition tasks.

### 4.6. Performance of the Proposed Method in Different Environments

In practical applications, obtaining real ground-truth images for comparison with the restoration results can be challenging or costly, making it difficult to determine whether the best results have been achieved. Therefore, this study further explored the performance of HEDIR-CNN in various real-world environmental conditions without the availability of ground-truth images. The goal was to input images captured under different environmental conditions into the designed CNN model, focusing on achieving optimal restoration performance for images with significant light variations.

In cases without ground-truth images, this study employed the No-Reference Image Quality Evaluator (NIQE) [30] and the Blind/Reference less Image Spatial Quality Evaluator (BRISQUE) [31] as quality metrics to assess restoration quality. The source code provided by both evaluators was used to calculate quality metrics. NIQE is a blind image quality assessment metric designed to evaluate the quality of an image without reference to real images or subjective human assessment. It is based on measuring the deviation of image statistical regularities from natural images to assess image quality; a smaller NIQE value indicates better image quality. Similarly, BRISQUE does not require a reference to the original image and only needs to evaluate the output image. Thus, BRISQUE is suitable for evaluating image quality when the original image is unavailable. The smaller the BRISQUE value, the better the image quality. Table 7 shows the image quality enhancement before and after using the proposed HEDIR-CNN. The NIQE and BRISQUE values of the restored image were improved by at least 25%.

## 5. Conclusions

This study aims to develop a supervised CNN architecture for HEDIR, which aims to restore images with details lost owing to strong light variation in the environment. To meet the requirement of a lightweight network, the proposed architecture is based on U-Net as the basic model, with a series of modifications and the incorporation of suitable network modules, including inception-like blocks, dual attention units, SKFUs, and denoising blocks. The inception-like block is an important component in the proposed architecture, which extracts features with different receptive fields to enhance the restoration effect. By replacing the conventional 5 × 5, 7 × 7, and 9 × 9 convolution filters with a series of cascaded 3 × 3 convolution filters, a wider receptive field is obtained while reducing the computational complexity during network training, thus improving the efficiency of the network.

The proposed HEDIR-CNN improves direct concatenation by introducing DAUs and SKFUs. This mechanism facilitates information sharing with features in both spatial and channel dimensions, using attention coefficients to adjust feature map gains and reduce attention to irrelevant regions. Furthermore, it aggregates and selects information from different layers to adjust the receptive field and enhance the model’s perception of global and local features in the image. An image denoising block is applied to reduce the noise in the image, thereby increasing the clarity and details, which contributes to object recognition and further enhances the image restoration results.

BAID’s experimental results demonstrated that the proposed HEDIR-CNN significantly improved the image quality metrics, including PSNR, SSIM, NIQE, and BRISQUE values. The HEDIR-CNN model proposed in this study consistently exhibited excellent restoration performance in various scenarios. The results will benefit further application to visual simultaneous localization and mapping and visual driving assistant systems.

## Figures and Tables

**Table 2:** The number of images in BAID for different scenes [16].

Category		Images
Outdoor	People	975
	Vehicle	186
	Building	325
	Plant	226
	Sculpture	177
Indoor	People	390
	Plant	195
	Furniture	262
	Ornament	264

**Table 3:** Ablation study.

	ILB	OAG	DAU	SKFF	DB
Model 1	-	-	-	-	-
Model 2	○	-	-	-	-
Model 3	○	○	-	-	-
Model 4	○	-	○	-	-
Model 5	○	-	○	○	-
Model 6 (proposed)	○	-	○	○	○

(ILB: Inception-like Block; OAG: Original Attention Gate; DAU: Dual Attention Unit; SKFF: Selective Kernel Feature Fusion; DB: Denoising Block).

**Table 4:** The network module ablation experiment was conducted using the BAID test dataset.

Methods	PSNR	SSIM	Trainable Parameters
Model 1	21.588	0.8742	442.083 k
Model 2	23.745	0.9071	174.171 k
Model 3	23.756	0.9053	185.089 k
Model 4	25.843	0.918	609.786 k
Model 5	26.239	0.9193	595.452 k
Model 6 (proposed)	27.042	0.9245	1.735 M

**Table 5:** Results of image restoration evaluation on the BAID test dataset.

Method	PSNR (% inc)	SSIM (% inc)
Backlit image enhancement [8]	17.96 (50.56)	0.86 (8.14)
Restoration of backlit images [7]	17.16 (57.58)	0.82 (13.41)
ExCNet [15]	19.31 (40.03)	0.90 (3.33)
Proposed Method	27.04	0.93
$Inc(\%)=\frac{\mathrm{ratio}\;\mathrm{by}\;\mathrm{the}\;\mathrm{proposed}\;-\;\mathrm{ratio}\;\mathrm{by}\;\mathrm{comparative}\;}{\mathrm{value}\;\mathrm{by}\;\mathrm{comparative}}\times 100$		

**Table 6:** Detected object count before and after restoration.

Object	Before	After
Person	8	13
Umbrella	0	1
Handbag	0	1
Sports ball	0	1

**Table 7:** NIQE and BRISQUE values before and after image restoration by the proposed HEDIR-CNN.

Image with High Exposure Difference [7]	NIQE		BRISQUE	
	Before	After	Before	After
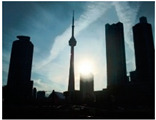	4.32	2.78	23.00	16.49
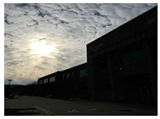	3.43	2.57	22.02	11.66

## Data Availability

Data are contained within the article.

## References

[1] Mishra B., Garg D., Narang P., Mishra V. (2020). Drone-surveillance for search and rescue in natural disaster. Comput. Commun..

[2] Mogili U.M.R., Deepak B.B.V.L. (2018). Review on Application of Drone Systems in Precision Agriculture. Procedia Comput. Sci..

[3] Howard J., Murashov V., Branche C.M. (2018). Unmanned aerial vehicles in construction and worker safety. Am. J. Ind. Med..

[4] Ma’Sum M.A., Arrofi M.K., Jati G., Arifin F., Kurniawan M.N., Mursanto P., Jatmiko W. Simulation of intelligent unmanned aerial vehicle (UAV) for military surveillance. Proceedings of the 2013 International Conference on Advanced Computer Science and Information Systems (ICACSIS).

[5] Wei C., Wang W., Yang W., Liu J. (2018). Deep Retinex Decomposition for Low-Light Enhancement. arXiv.

[6] Zamir S.W., Arora A., Khan S., Hayat M., Khan F.S., Yang M.-H., Shao L. (2020). Learning enriched features for real image restoration and enhancement. Proceedings of the Computer Vision–ECCV 2020: 16th European Conference.

[7] Zhenhao L., Xiaolin W. (2018). Learning-Based Restoration of Backlit Images. IEEE Trans. Image Process..

[8] Wang Q., Fu X., Zhang X.-P., Ding X. A fusion-based method for single backlit image enhancement. Proceedings of the 2016 IEEE International Conference on Image Processing (ICIP).

[9] Buades A., Lisani J.L., Petro A.B., Sbert C. (2020). Backlit images enhancement using global tone mappings and image fusion. IET Image Process..

[10] Mertens T., Kautz J., Van Reeth F. Exposure fusion. Proceedings of the 15th Pacific Conference on Computer Graphics and Applications (PG’07).

[11] Li X.P., Lu Q.Y., Fan C., Zhao C., Zou L., Yu L. (2024). Generalizing event-based HDR imaging to various exposures. Neurocomputing.

[12] Jia X.Y., Lin Q.W.N., Ding W.P. (2024). An ultra-high-definition multi-exposure image fusion method based on multi-scale feature extraction. Appl. Soft. Comput..

[13] Lin X., Tan P.W., Wang Z.K., Ma L.Z., Li Y. (2024). DDFL: Dual-Domain Feature Learning for nighttime semantic segmentation. Displays.

[14] Yaqoob M., Ishaq M., Ansari M.Y., Konagandla V.R.S., Tamimi T.A., Tavani S., Corradetti A., Seers T.D. (2024). GeoCrack: A High-Resolution Dataset For Segmentation of Fracture Edges in Geological Outcrops. Sci. Data.

[15] Zhang L., Zhang L., Liu X., Shen Y., Zhang S., Zhao S. Zero-shot restoration of back-lit images using deep internal learning. Proceedings of the 27th ACM International Conference on Multimedia.

[16] Lv X., Zhang S., Liu Q., Xie H., Zhong B., Zhou H. (2022). BacklitNet: A dataset and network for backlit image enhancement. Comput. Vis. Image Underst..

[17] Ronneberger O., Fischer P., Brox T. (2015). U-net: Convolutional networks for biomedical image segmentation. Proceedings of the Medical Image Computing and Computer-Assisted Intervention–MICCAI 2015: 18th International Conference.

[18] Tsai C.-Y., Chen C.-L. (2022). Attention-Gate-Based Model with Inception-like Block for Single-Image Dehazing. Appl. Sci..

[19] Odena A., Dumoulin V., Olah C. (2016). Deconvolution and Checkerboard Artifacts. Distill.

[20] Szegedy C., Vanhoucke V., Ioffe S., Shlens J., Wojna Z. Rethinking the inception architecture for computer vision. Proceedings of the IEEE Conference on Computer Vision and Pattern Recognition.

[21] Oktay O., Schlemper J., Folgoc L.L., Lee M., Heinrich M., Misawa K., Mori K., McDonagh S., Hammerla N.Y., Kainz B. (2018). Attention u-net: Learning where to look for the pancreas. arXiv.

[22] Jian B.-L., Tsai C.-S., Kuo Y.-C., Guo Y.-S. (2019). An image vision and automatic calibration system for universal robots. J. Low Freq. Noise Vib. Act. Control.

[23] Gurrola-Ramos J., Dalmau O., Alarcon T.E. (2021). A Residual Dense U-Net Neural Network for Image Denoising. IEEE Access.

[24] Zhao H., Gallo O., Frosio I., Kautz J. (2017). Loss Functions for Image Restoration With Neural Networks. IEEE Trans. Comput. Imaging.

[25] Wang Z., Bovik A.C., Sheikh H.R., Simoncelli E.P. (2004). Image quality assessment: From error visibility to structural similarity. IEEE Trans. Image Process..

[26] Liu Y., Zhao G. (2018). Pad-net: A perception-aided single image dehazing network. arXiv.

[27] Avcibas I., Sankur B., Sayood K. (2002). Statistical evaluation of image quality measures. J. Electron. Imaging.

[28] Kingma D.P., Ba J. (2014). Adam: A method for stochastic optimization. arXiv.

[29] Bochkovskiy A., Wang C.-Y., Liao H.-Y.M. (2020). Yolov4: Optimal speed and accuracy of object detection. arXiv.

[30] Mittal A., Soundararajan R., Bovik A.C. (2013). Making a “Completely Blind” Image Quality Analyzer. IEEE Signal Process. Lett..

[31] Mittal A., Moorthy A.K., Bovik A.C. Blind/referenceless image spatial quality evaluator. Proceedings of the 2011 Conference record of the Forty Fifth Asilomar Conference on Signals, Systems and Computers (ASILOMAR).

